# The Two-Way Interaction between the Molecules That Cause Vaginal Malodour and Lactobacilli: An Opportunity for Probiotics

**DOI:** 10.3390/ijms222212279

**Published:** 2021-11-13

**Authors:** Scarlett Puebla-Barragan, Polycronis Paul Akouris, Kait F. Al, Charles Carr, Britney Lamb, Mark Sumarah, Charlotte van der Veer, Remco Kort, Jeremy Burton, Gregor Reid

**Affiliations:** 1 Canadian Centre for Human Microbiome and Probiotics, Lawson Health Research Institute, 268 Grosvenor Street, London, ON N6A 4V2, Canada; pakouris@uwo.ca (P.P.A.); kal@uwo.ca (K.F.A.); charlie.carr@mail.utoronto.ca (C.C.); blamb5@uwo.ca (B.L.); Jeremy.Burton@lawsonresearch.com (J.B.); gregor@uwo.ca (G.R.); 2 Departments of Microbiology and Immunology, Surgery, and Biochemistry, Western University, London, ON N6A 4V2, Canada; 3 Agriculture and Agri-Food Canada, London, ON N5V 4T3, Canada; mark.sumarah@canada.ca; 4 Department of Infectious Diseases, Public Health Service (GGD), Nieuwe Achtergracht 100, 1018 WT Amsterdam, The Netherlands; cvanderveer@mlw.mw; 5 Department of Molecular Cell Biology, Faculty of Science, O2 Lab Building, Vrije Universiteit Amsterdam, De Boelelaan 1108, 1081 HZ Amsterdam, The Netherlands; r.kort@vu.nl; 6 ARTIS-Micropia, Plantage Kerklaan 38-40, 1018 CZ Amsterdam, The Netherlands

**Keywords:** vaginal malodour, dysbiosis, probiotics, lactobacilli, vaginal microbiota, biogenic amines

## Abstract

Vaginal malodour is a sign of dysbiosis. The biogenic amines (BAs) cadaverine, putrescine and tyramine are known to be causative compounds. Recent reports suggest these compounds produced by pathogens might have a role beyond causing malodour; namely inhibiting the growth of lactobacilli bacteria that are crucial in the maintenance of vaginal homeostasis. The aim of this study was to identify whether certain lactobacilli strains could reduce BAs and to evaluate how *Lactobacillus* species were affected by these compounds. Using LC–MS and HPLC-UV, five *Lactobacillus crispatus* strains were identified as being capable of significantly reducing BAs from the media under in vitro conditions. Through 16S rRNA gene sequencing of vaginal swabs exposed to Bas, cadaverine was found to reduce the relative abundance of lactobacilli. When *L. crispatus* was exposed to media supplemented with BAs with an HCl adjusted lower pH, its growth was enhanced, demonstrating the relevance of the maintenance of an acidic vaginal environment. If strains are to be developed for probiotic application to alleviate bacterial vaginosis and other conditions affecting large numbers of women worldwide, their ability to adapt to Bas and regulate pH should be part of the experimentation.

## 1. Introduction

The biogenic amines (BAs) cadaverine, putrescine, and tyramine, have been correlated with malodour in the urogenital tract of women with dysbiosis. Patients with conditions such as bacterial vaginosis (BV) and urinary tract infection (UTI) suffer from malodour, negatively impacting their quality of life [1,2]. Unfortunately, the gold standard treatment for both conditions is a course of antibiotics. However, these do not target malodour, raising the concept that conjoint treatment could be developed.

Probiotics, defined as ‘live microorganisms that, when administered in adequate amounts, confer a health benefit on the host’ [3,4], have been used for decades to help manage and prevent dysbiosis of the female urogenital tract. Studies primarily using *Lacticaseibacillus rhamnosus* GR-1 (LGR-1) and *Limosilactobacillus reuteri* RC-14 (LRC-14) have shown benefits in regulating homeostasis [5,6,7,8]. Indeed, LGR-1 is the most widely studied probiotic for urogenital health [5,6], and its genome indicates that it can thrive in the vaginal environment [5,6]. When used in conjunction with LRC-14 it can decrease recurrence of BV, UTI and effectively inhibit the yeast *Candida albicans*, which is one of the most frequent vaginal pathogens [7,8,9]. Additionally, the combination taken orally during and following antibiotic treatment aids in the replenishment of indigenous species such as *Lactobacillus crispatus*, which are predominant in most healthy vaginas [10]. This suggests that lactobacilli-based probiotics could be used, in combination with antibiotics, to address malodour. Nonetheless, not all probiotics are the same, and their health benefits must be well documented. As such, it was our goal to identify which strains could potentially aid in the reduction of malodorous compounds and to provide a better understanding of the mechanisms involved.

Previous studies have suggested that BAs are produced by some pathogens and they act as virulence factors that increase the urogenital pH, reduce production of lactic acid, and lower the abundance of lactobacilli [11,12]. Previously, several strains of *L. crispatus* have exhibited the potential to degrade BAs [13]. In the present study, five clinical isolates of *L. crispatus* were tested for their ability to reduce BAs, along with the type-strain *L. crispatus* ATCC 33820, LGR-1, and LRC-14. The relationship between these malodorous compounds and lactobacilli survival was also examined. We hypothesized that the interaction between lactobacilli and BAs is key for the maintenance of vaginal homeostasis. The long-term goal is to determine if probiotics represent a novel approach to manage urogenital dysbiosis and malodour. 

## 2. Results

### 2.1. Lactobacilli Growth in Biogenic Amines

**Figure 1:**
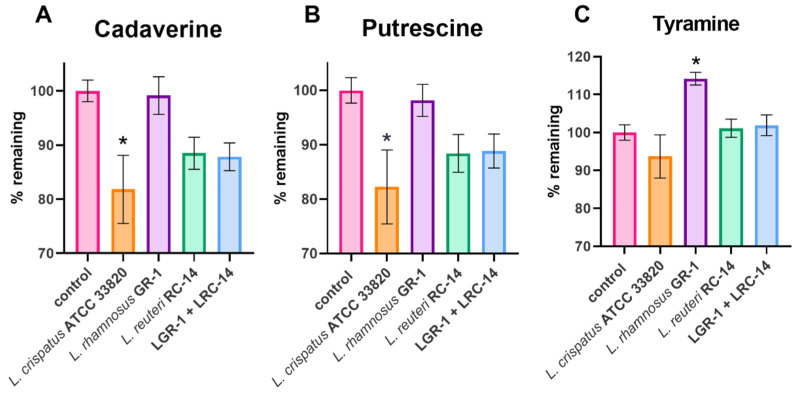
Metabolism of biogenic amines by different lactobacilli strains. Control consisted of media supplemented with each BA of interest. Bar plots show the remaining concentration of (**A**) cadaverine, (**B**) putrescine, and (**C**) tyramine, after 24 h of incubation with each strain of interest. Data are presented as means of 4 independent experiments ±95% confidence intervals (CI). One-way ANOVA with the Dunnet correction for multiple comparisons was used to calculate statistical significance, * *p* ≤ 0.05).

When grown in VDMP supplemented with 100 µg/mL of cadaverine, putrescine, or tyramine as independent experiments, only *L. crispatus* ATCC 33820 showed a statistically significant reduction of cadaverine and putrescine. Strain LGR-1 produced a significant amount of tyramine when grown by itself, but this was tempered when LRC-14 was added. LRC14 showed a decreasing trend in the amounts of cadaverine and putrescine, but statistical significance was not reached. Results are summarized in Figure 1. 

**Figure 2:**
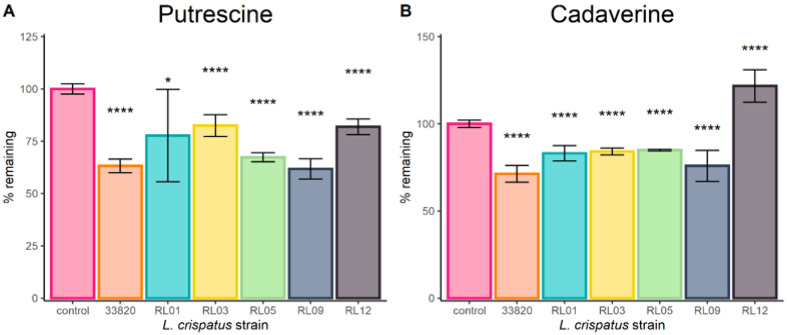
Metabolism of biogenic amines by different *L. crispatus* strains in amine VDMP. Control consists of amine VDMP only (no bacteria). Bar plots show the remaining concentration of (**A**) cadaverine and (**B**) putrescine, after 24 h of incubation with each strain of interest, One-way ANOVA with the Dunnet correction for multiple comparisons was used to calculate statistical significance (* *p* ≤ 0.05 and **** *p* ≤ 0.0001). Data are presented as means of 3 independent experiments with two technical replicates each ±95% CI.

Figure 2 shows that *L. crispatus* clinical isolates could significantly reduce the concentration of putrescine and cadaverine when supplemented with 200 µg/mL each, with the exception of strain RL12, which increased the amount of cadaverine significantly. Furthermore, all strains completely removed tyramine from the media. No biogenic amines were detected in any of the negative controls. 

**Figure 3:**
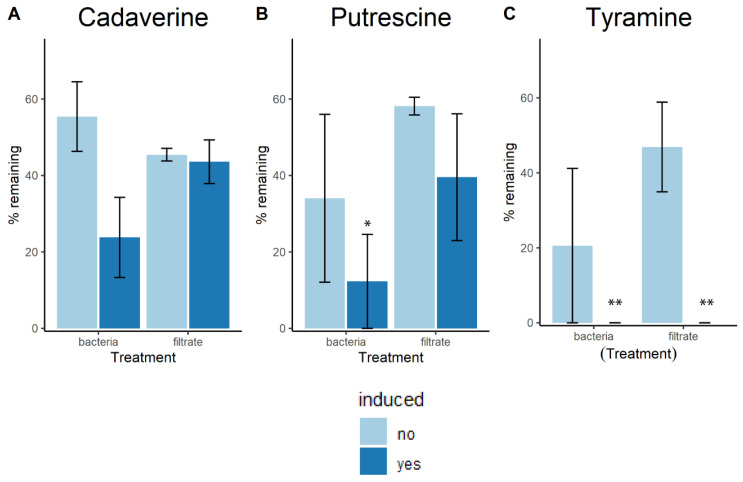
Biogenic amines exposure assay. Bar plots show the remaining concentration of (**A**) cadaverine, (**B**) putrescine, and (**C**) tyramine, after incubation with each amine of interest. One-way ANOVA with the Tukey post hoc test for multiple comparisons was used to calculate statistical significance (* *p* ≤ 0.05 and ** *p* ≤ 0.01). Data are presented as means of 3 independent experiments two technical replicates each ±95% CI.

When amine-reduction assays were carried out using cultures of *L. crispatus* ATCC 33820 that had been originally exposed to putrescine, cadaverine, and tyramine (i.e., ‘induced’ cultures), all preparations from either whole cells or supernatants reduced the amount of all tested amines below 60%. There was no statistically significant difference between induction and treatments for reduction ability of cadaverine. In the case of putrescine, ‘induced’ bacterial cultures showed further amine depletion than other treatments. Both the whole cells and the supernatant of ‘induced’ cultures reduced significantly more tyramine. Results are presented in Figure 3. 

Due to the physicochemical properties of TMA, none of the analytical methods (i.e., LC–MS/MS and HPLC-UV) were suitable for detecting this compound at relevant concentrations. Therefore, it was excluded.

### 2.2. Effect of Biogenic Amines and pH on the Growth of Lactobacillus Crispatus 

Originally, the pH of non-amine-supplemented VDMP (control) was 6.71 and that of BA media was 7.04. An additional set of each media was prepared with adjusted pH that corresponded to its counterpart (i.e., control at 7.04 and BA-media at 6.71). At the end of the incubation period all treatments reached a final pH of 4.50. 

**Figure 4:**
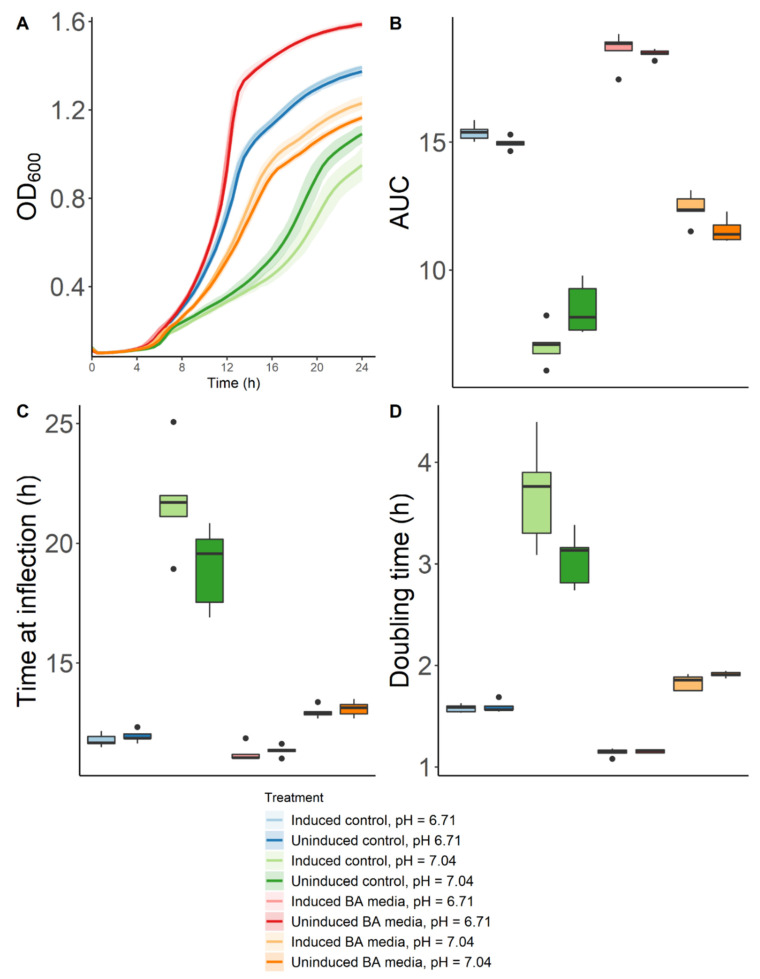
Effect of biogenic amines and pH on the growth of *L. crispatus* ATCC 33820. Data are presented as the means of 5 independent experiments. (**A**) Bacterial growth curves measured by absorbance at a wavelength of 600 nm. (**B**) Logistic areas under the curve. (**C**) Time at inflection. (**D**) Doubling times.

Results showed that both ‘induced’ and ‘uninduced’ cultures had the most and fastest growth when placed in BA–VDMP that had been adjusted to a lower pH (Figure 4). They were followed by ‘induced’ and ‘uninduced’ cultures grown in VDMP at its original pH (6.71). Next, the bacteria were grown in BA–media at its original pH (7.04); ‘induced’ cultures showed better growth than their ‘uninduced’ counterparts. The slowest and lowest growths were observed for strains in VDMP at an adjusted higher pH (7.04). Statistical analyses of different relevant growth parameters between groups are summarized in Supplementary Tables S1–S8.

**Figure 5:**
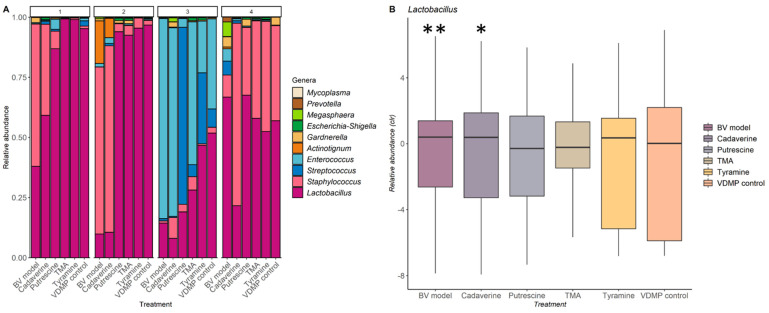
Impact of biogenic amines on the vaginal microbiota. (**A**) Bar plot of relative abundances. Each colour represents a genus and top numbers indicate each subject from which the sample originated. *X*-axis denotes which treatment each swab underwent. (**B**) Relative abundances of *Lactobacillus* spp. in different media (centered log ratios (CLR) are plotted). (* *p* ≤ 0.05 and ** *p* ≤ 0.01).

Analyses of the impact of exposure to biogenic amines on vaginal microbiota are shown in Figure 5. Results revealed that *Lactobacillus* abundance was significantly reduced when cultures were exposed to 200 µg/mL of cadaverine or to media supplemented with biogenic amines at similar concentrations to those found in vaginal secretions during BV [11,12].

## 3. Discussion

Probiotic strains LRC-14 and LGR-1 showed some reduction of cadaverine and putrescine concentrations (both individually and as a coculture). Interestingly, LGR-1 significantly increased tyramine; however, when cocultured with LRC-14 this effect was not observed. These two strains are administered in combination, and these findings suggests a further benefit of this cooperation [14,15,16,17,18]. 

We previously characterized several clinical *L. crispatus* strains in terms of their potential to degrade and/or produce biogenic amines, while all of them have the genes for both functions, only a few were able to reduce the amount of amines in growth media. This formed the basis for selecting the strains for the present study [13]. Of significant note, *L. crispatus* ATCC 33820 and five vaginal *L. crispatus* isolates showed statistically significant reduction of putrescine, cadaverine and tyramine within 24 h. The exception was strain RL12 which increased tyramine. Cultures of the type strain which had been either exposed (‘induced’) or not (‘uninduced’) to biogenic amines prior to the experiment resulted in reduced concentration of BAs from the media. Furthermore, bacteria originating from ‘induced’ cultures had a higher capacity to reduce the concentration of amines, better resistance to high pH, and they grew better in the presence of amines. Similarly, filtered supernatants from ‘uninduced’ and especially ‘induced’ cultures were able to reduce the concentration of amines. It is unclear if this adaptation is pH- or metabolite-mediated. Previous studies have shown that the expression of the arginine deiminase pathway (which plays a major role in the metabolism of cadaverine and putrescine [13]) is pH-dependent in other *Lactobacillus* species [19]. Future studies should focus on global metabolomics and transcriptomic analyses that might identify which metabolic pathways are different between both groups and could therefore be causing these differences. 

Interestingly, as shown in Figure 4A, when the high pH caused by BA was adjusted, *L. crispatus* ATCC 33820 grew better than in the medium alone. This novel finding suggests that under low pH conditions, lactobacilli can use biogenic amines as nutrient sources. This raises the question of whether in some women who experience malodour that self-resolves, the indigenous *L. crispatus* have been able to adapt and reassert themselves. 

It has been previously shown that when growth medium is acidified via the addition of HCl, as was the case in our study, the specific growth rate of lactobacilli and the production of lactic acid are slowed down [20]. However, the presence of metabolizable sugars has a protective role and enhances survival in *L. rhamnosus* GG [21]. Our results suggest that this could also be true of biogenic amines, which become bioavailable energy sources at lower pH. Further studies are required using different minimal growth media supplemented with BAs to predict the metabolic pathways in which the biogenic amines are being used by the bacteria. In addition, this could verify if this effect is translatable to various lactobacilli strains, or, if it is a phenomenon specific for *L. crispatus* spp. Specifically, transcriptomic and metabolomic studies are warranted to better understand the metabolic adaptations that might occur as responses to changes in the pH of the environment. 

The buffering capacity of vaginal fluids has been previously suggested as an indicator of reproductive health [22]. Thus, approaches that aid in the maintenance of an acidic environment, such as the use of prebiotics [23], are highly relevant to regulate the concentration of biogenic amines, thereby preventing dysbiosis and malodour. 

Previous observations determined that biogenic amine exposure slowed down and reduced the growth capacity of lactobacilli [12]. However, we identified that this inhibitory effect can be reversed depending on the pH conditions. This might explain why some lactobacilli strains can produce small amounts of biogenic amines to support the growth of others, thereby maintaining an acidic environment that prevents the proliferation of pathogenic bacteria [13]. A limitation of our study is that we cannot infer causality from our observations. In the future, multiomics analyses should further characterize the phenomena we observed and aim to better understand the mechanisms behind them.

The microbiota composition study of vaginal samples showed that at concentrations of biogenic amines like those found in the vaginal fluids of patients with BV, the relative abundance of *Lactobacillus* spp. is reduced and associated with an increase in pH. This, in turn, allows for further proliferation of pathogens towards a state of dysbiosis. Furthermore, only media containing cadaverine had the ability to significantly decrease the proportion of *Lactobacillus* spp., suggesting that this compound plays a key role in the infection process and onset of malodour. The impact of other amines is not clear and should be further explored with larger sample sizes. 

Overall, these findings provide a strong rationale for assessing candidate probiotic lactobacilli strains for their capacity to degrade biogenic amines and regulate pH. This study highlights the relevance of maintaining a low pH in the vaginal environment, since this can turn biogenic amines from inhibitory compounds into energy sources that support the growth of lactobacilli. 

## 4. Materials and Methods

### 4.1. Lactobacillus Crispatus Clinical Strains

The *L. crispatus* strains used in this study were selected based on their previously determined ability to reduce the concentration of BAs [13] and their genetic potential to degrade BAs. Strains were provided by van der Veer et al. [24] with all material approved by each participant with written consent and approval from the ethics review board of the Academic Medical Center (AMC), University of Amsterdam, The Netherlands. They originated from vaginal swabs obtained from women attending for a health check at the Sexually Transmitted Infections Clinic in Amsterdam, The Netherlands. The samples came from two groups of women: one with healthy vaginal microbiota (based upon Nugent score 0–3), or those with dysbiosis (Nugent score 7–10). The swabs were plated on modified trypticase soy agar and incubated anaerobically at 37 °C for 24 h. Next, single colonies underwent 16S rRNA gene sequencing for identification purposes. The strains were then cryopreserved and stored at −80 °C in vaginally defined medium plus peptone (VDMP) [25]. Additional strains were obtained in London, Ontario, Canada from four healthy premenopausal women aged between 25 and 30 years old who consented to partake in the study, approved by the Health Sciences Research Ethics Board at The University of Western Ontario (File number 106089, original approval date 13 January 2015) [23]. The subjects self-swabbed their lateral vaginal walls using sterile Dacron swabs. Vaginal pH was confirmed to be approximately 4.5 by using pHem-Alert applicator keys (Gynex Corporation, Redmond, WA, USA). Swabs were Gram-stained (Becton, Dickinson & Company, Franklin Lakes, NJ, USA) and scored 0–3 using the Nugent system [26,27].

### 4.2. Biogenic Amine Reduction Experiments

An amine-reduction assay using LGR-1, LRC-14, and *L. crispatus* ATCC 33820 was performed, along with a polymicrobial culture containing LGR1 and LRC-14. Four individual colonies of each organism were grown in de Man–Rogosa–Sharpe (MRS) broth medium (VWR) at 37 °C anaerobically for 24 h. Next, 10 µL of the initial liquid cultures was grown for 24 h anaerobically at 37 °C in 10 mL of VDMP supplemented with 100 µg/mL of either cadaverine, putrescine, and tyramine (Sigma Aldrich, St. Louis, MO, USA) or a control of each specific amine media without bacteria to control for aerial effect of the volatile compounds. These were subsequently analyzed using liquid chromatography–mass spectrometry (LC-MS/MS).

To test their ability to degrade amines at similar concentrations to those in vaginal dysbiosis [28], three individual colonies of five *L. crispatus* strains (i.e., RL01, RL03, RL05, RL09, and RL12) as well as the type strain (ATCC 33820) were grown in MRS medium at 37 °C anaerobically for 24 h. Then, 10 µL was subcultured in 2 mL of either VDMP or amine–VDMP (200 µg/mL of cadaverine, 200 µg/mL of putrescine, and 200 µg/mL of tyramine) (Sigma Aldrich, St. Louis, MO, USA) and grown anaerobically at 37 °C for 24 h. The control consisted of amine–VDMP or VDMP with no bacteria. These samples were analysed using high performance liquid chromatography coupled to an ultraviolet detector (HPLC–UV). 

### 4.3. Induction Assays

To identify whether *L. crispatus* undergoes adaptation after exposure to BAs, five individual colonies of *L. crispatus* ATCC 33820 were grown in 2 mL of amine–VDMP anaerobically at 37 °C for 24 h, then 10 µL was inoculated into 2 mL of amine–VDMP under the same conditions; these were deemed the ‘induced’ group. Meanwhile, the ‘uninduced’ group was derived from 5 individual colonies of *L. crispatus* ATCC 33820 grown in 2 mL of VDMP anaerobically at 37 °C for 24 h, and subsequently subcultured in amine–VDMP. Next, HPLC–UV analysis was performed.

To assess whether the metabolites produced by *L. crispatus* had the ability to degrade BAs, the spent media of the original liquid cultures was filter sterilized (0.22 µm) and incubated under the same conditions as the previous samples in amine–VDMP. These groups were designed ‘induced’ and ‘uninduced’ filtrates. 

The effect of pH on the tolerance to amines of ‘induced’ and ‘uninduced’ cultures was measured by inoculating a 1:100 dilution of the liquid cultures in 240 µL of BA–media (amine–VDMP + 50 µg/mL of TMA). The control consisted of VDMP only (*n* = 5). The pH was adjusted using either NaOH or HCl. Absorbance at 600 nm was measured every 30 min over 24 h in an automatic plate reader. Finally, pH was measured using pH strips (Eorta). The R package growthcurveR [29] was used to model the bacterial growth kinetics and to calculate relevant growth parameters (i.e., maximum growth capacity, area under the curve, time at inflection point, and doubling time). 

Data analysis was performed based on linear models generated for each growth parameter. Families were defined as either induced or uninduced. Within each family, multiple comparisons and control for type I error were performed using Tukey’s post hoc test (Supplementary Tables S1, S3, S5 and S7). Next, individual contrasts between both families at every pH were performed (Supplementary Tables S2, S4, S6 and S8).

### 4.4. LC–MS/MS Protocol

After incubation, 250 µL of each liquid culture was aliquoted and diluted 1:3 with pure methanol then vortexed and incubated on ice for 30 min, followed by centrifugation at 16,000× *g* for 10 min. Supernatants were filtered into vials for analysis (0.22 µm PTFE syringe filters) which was carried out using a Q-Exactive Quadrupole Orbitrap MS, coupled to an Agilent 1290 HPLC system with an Agilent HILIC-Z (2.1 × 100 mm, 2.7 µm). The mobile phase A consisted of 20 mM ammonium formate in water, and phase B was 20 mM ammonium formate in 90% acetonitrile. Analytes were eluted with the following gradient: 0 min, 100% B; 0.5 min, 100% B; 5.3 min, 80% B; 9.5 min, 30% B; 13.5 min, 30% B; 14.5 min 100% B; and 16.5 min, 100% B. Conditions for heated electrospray ionization (HESI) were as follows: HESI(+) capillary voltage, capillary voltage, 3.5 kV; capillary temperature, 250 °C; sheath gas, 30.00 units; auxiliary gas, 8.00 units; probe heater temperature, 450 °C; S-Lens RF level, 60.00. The instrument was operated with a top-3 data-dependent acquisition protocol with a full MS scan in the mass range of m/z 1.2 and collision energy of 28. Authentic standards were used for calibration curves (cadaverine, putrescine, and tyramine), acquired from Sigma-Aldrich (St. Louis, MO, USA). Compound identification and quantification was performed using the software Xcalibur (Thermo Fisher Scientific, Waltham, MA, USA).

### 4.5. HPLC–UV Analysis

Bacterial cultures and their supernatants were prepared by diluting them 1:10 with water, incubating them on ice for 30 min and centrifuging at 16,000× *g* for 10 min. Samples were prepared for derivatization by adding 250 µL of 0.5 KH_2_PO_4_ buffer (pH = 11) and 10 µL of 1 M NaOH. Next, 500 µL of p-toluenesulfonyl chloride (10 mg/mL, Sigma Aldrich, St. Louis, MO, USA) was added and samples were incubated at 56 °C for 10 min. The reaction was stopped with the addition of 50 µL of 1 M HCl and samples were filtered into vials (0.45 µL PTFE syringe filters) [30].

Analysis was carried out using a mobile phase in isocratic mode, composed of 60% acetonitrile and 40% HPLC-grade water (Thermo Fisher Scientific, Waltham, MA, USA). The flow rate was 1 mg/mL. Authentic standards for putrescine, cadaverine, and putrescine were used, and α-aminobutyric acid was used as an internal standard, all acquired from Sigma-Aldrich. Analyte identification and quantification was performed using the Chemstation software B.04.03 (Agilent, Santa Clara, CA, USA).

### 4.6. Analysis of the Impact of Biogenic Amines on the Vaginal Microbiota

To assess the impact that biogenic amines have on the vaginal microbiota, an adaptation of the in vitro, polymicrobial, vaginal culturing model described previously was used [23]. Briefly, bacteria collected from vaginal swabs were grown anaerobically for 36 h in different media: VDMP supplemented with either 200 µg/mL of cadaverine, 200 µg/mL of putrescine, 200 µg/mL of tyramine, or 50 µg/mL of TMA, BA–VDMP, and VDMP. Different media were prepared to properly evaluate the effect of each amine alone and as a combination. The concentrations of amines were selected based on what is normally found in vaginal fluids of patients with BV. 

Microbiota analysis was performed by amplification of the V4 region of 16S ribosomal RNA, which was then sequenced using the Illumina MiSeq (San Diego, CA, USA) to detect shifts in microbial abundance [31]. Earth Microbiome universal primers, 515F and 806R, were used for PCR amplification. Primers consisted of an Illumina adapter and four random nucleotides, one of 24 unique 12-mer barcodes, and the corresponding annealing primer [32]. A Biomek^®^ 3000 Laboratory Automation Workstation (Beckman-Coulter, Mississauga, ON, Canada) was used for PCR set-up. Amplification was performed in an Eppendorf thermal cycler (Eppendorf, Mississauga, ON, Canada) with an initial rise in temperature of 95 °C, then 25 cycles of one minute each at 95 °C, 52 °C, and 72 °C. Purified amplicons were then paired-end sequenced with 250 cycles on an Illumina MiSeq platform (San Diego, CA, USA) [32].

Data were exported as raw fastq files. Quality control was performed following the DADA2 pipeline [32]. Taxonomy was assigned using the SILVA (c132) training set, and reads corresponding to Eukaryota, Mitochondria, and Chloroplast were removed [32]. Amplicon sequence variants (SVs) present at ≥1% relative abundance were maintained. Downstream analysis was performed with the ALDEx2 [33,34,35], Vegan [36], rstatix [37], and emmeans [38] R packages.

### 4.7. Statistical Analyses

All data analyses were performed using RStudio. One-way analysis of variances (ANOVAs) and all post hoc tests were conducted with the R packages rstatix v0.7.0 [37]. To correct heteroscedasticity, marginal means were used and the matrix of covariance was adjusted. This was performed using the R packages emmeans v1.6.0 [38] and sandwich v3.0-1 [39]. Figure 1 was created using the software GraphPad Prism v.8. The rest of the figures were made with the R package ggplot2 [40].

## Data Availability

The data for this study have been deposited in the European Nucleotide Archive (ENA) at EMBL-EBI under accession number PRJEB48740.

## Supplementary Materials

The following are available online at https://www.mdpi.com/article/10.3390/ijms222212279/s1. Table S1: Effect of biogenic amine supplemented media on maximum possible population (K). Table S2: Effect of previous amine exposure on maximum possible population (K). Table S3: Effect of biogenic amine supplemented media on time at inflection. Table S4: Effect of previous amine exposure on time at inflection. Table S5: Effect of amine supplemented media on doubling time. Table S6: Effect of previous amine exposure on doubling time. Table S7: Effect of amine supplemented media on logistic area under the curve. Table S8: Effect of previous amine exposure on logistic area under the curve.
