# Supplementary material for: The Two-Way Interaction between the Molecules That Cause Vaginal Malodour and Lactobacilli: An Opportunity for Probiotics
Source: Int J Mol Sci. 2021 Nov 13;22(22):12279. doi: 10.3390/ijms222212279 (PMC8621103; doi:10.3390/ijms222212279)
Supplement: Supplementary file 1 [file ijms-22-12279-s001.zip › ijms-1420260-supplementary.pdf]

International Journal of *Molecular Sciences*

MDPI

Probiotics to reduce biogenic amines that cause urogenital malodour

Scarlett Puebla-Barragan [1,2,*], Polycronis Paul Akouris [1,2], Kait Al [1,2], Charles Carr [1,2], Britney Lamb [1,2], Mark Sumarah [3], Charlotte van der Veer [4], Remco Kort [5,6], Jeremy Burton [1,2] and Gregor Reid [1,2]

1 Canadian Centre for Human Microbiome and Probiotics, Lawson Health Research Institute, 268 Grosvenor Street, London, ON N6A 4V2, Canada; pakouris@uwo.ca (P.P.A.); kal@uwo.ca (K.A.); charlie.carr@mail.utoronto.ca (C.C.); blamb5@uwo.ca (B.L.); Jeremy.Burton@lawsonresearch.com (J.B.); gregor@uwo.ca (G.R.)

2 Departments of Microbiology and Immunology, and Surgery, Western University, London, ON N6A 4V2, Canada

3 Agriculture and Agri-Food Canada, London, ON N5V 4T3, Canada; mark.sumarah@canada.ca

4 Department of Infectious Diseases, Public Health Service (GGD), Nieuwe Achtergracht 100, 1018 WT Amsterdam, The Netherlands; cvanderveer@mlw.mw

5 Department of Molecular Cell Biology, Faculty of Science, O2 Lab Building, Vrije Universiteit Amsterdam, De Boelelaan 1108, 1081 HZ Amsterdam, The Netherlands; r.kort@vu.nl

6 ARTIS-Micropia, Plantage Kerklaan 38-40, 1018 CZ Amsterdam, The Netherlands

Table S1. Effect of biogenic amine supplemented media on maximum possible population (K).

Maximum possible population (K): Multiple comparisons
Induced vs uninduced samples

Treatment 1	Treatment 2	P-Value	Significance (α 0.05)
**Induced**			
Control (pH = 6.71)	Control (pH = 7.04)	0.012	*
Control (pH = 6.71)	BA media (pH = 6.71)	≤ 0.0001	****
Control (pH = 6.71)	BA media (pH = 7.04)	≤ 0.0001	****
Control (pH = 7.04)	BA media (pH = 6.71)	0.995	ns
Control (pH = 7.04)	BA media (pH = 7.04)	≤ 0.0001	****
BA media (pH = 6.71)	BA media (pH = 7.04)	≤ 0.0001	****
**Uninduced**			
Control (pH = 6.71)	Control (pH = 7.04)	0.067	ns
Control (pH = 6.71)	BA media (pH = 6.71)	≤ 0.0001	****
Control (pH = 6.71)	BA media (pH = 7.04)	≤ 0.0001	****
Control (pH = 7.04)	BA media (pH = 6.71)	0.415	ns
Control (pH = 7.04)	BA media (pH = 7.04)	≤ 0.0001	****
BA media (pH = 6.71)	BA media (pH = 7.04)	≤ 0.0001	****

Multiple comparisons between control group (non-supplemented VDMP media, original pH 6.71) and Biogenic amine (BA) media (original pH). An additional media of each was prepared and its pH adjusted to mimic its supplemented/non-supplemented counterpart. Induced samples were grown from cultures previously exposed to BAs and uninduced from cultures grown in non-supplemented VDMP.

Table S2. Effect of previous amine exposure on maximum possible population (K).

Maximum possible population (K): Multiple comparisons<br>Induced vs uninduced samples			
**Treatment 1**	**Treatment 2**	**P-Value**	**Significance (α 0.05)**
**Control (pH = 6.71)**			
Induced	Uninduced	0.217	ns
**Control (pH = 7.04)**			
Induced	Uninduced	0.387	ns
**BA media (pH = 6.71)**			
Induced	Uninduced	0.865	ns
**BA media (pH = 7.04)**			
Induced	Uninduced	0.001	**

Multiple comparisons between induced samples grown from cultures previously exposed to BAs and uninduced from cultures grown in non-supplemented VDMP. Cultures grown in (non-supplemented VDMP media, original pH 6.71) and biogenic amine (BA) media (original pH). An additional media of each was prepared and its pH adjusted to mimic its supplemented/non-supplemented counterpart.

Table S3. Effect of biogenic amine supplemented media on time at inflection.

Time at inflection: Multiple comparisons<br>Induced vs uninduced samples			
**Treatment 1**	**Treatment 2**	**P-Value**	**Significance (α 0.05)**
**Induced**			
Control (pH = 6.71)	Control (pH = 7.04)	≤ 0.0001	****
Control (pH = 6.71)	BA media (pH = 6.71)	0.086	ns
Control (pH = 6.71)	BA media (pH = 7.04)	≤ 0.0001	****
Control (pH = 7.04)	BA media (pH = 6.71)	≤ 0.0001	****
Control (pH = 7.04)	BA media (pH = 7.04)	≤ 0.0001	****
BA media (pH = 6.71)	BA media (pH = 7.04)	≤ 0.0001	****
**Uninduced**			
Control (pH = 6.71)	Control (pH = 7.04)	≤ 0.0001	****
Control (pH = 6.71)	BA media (pH = 6.71)	0.005	**
Control (pH = 6.71)	BA media (pH = 7.04)	≤ 0.0001	****
Control (pH = 7.04)	BA media (pH = 6.71)	≤ 0.0001	****
Control (pH = 7.04)	BA media (pH = 7.04)	≤ 0.0001	****
BA media (pH = 6.71)	BA media (pH = 7.04)	≤ 0.0001	****

Multiple comparisons between control group (non-supplemented VDMP media, original pH 6.71) and Biogenic amine (BA) media (original pH). An additional media of each was prepared and its pH adjusted to mimic its supplemented/non-supplemented counterpart. Induced samples were grown from cultures previously exposed to BAs and uninduced from cultures grown in non-supplemented VDMP.

Table S4. Effect of previous amine exposure on time at inflection**.**

Time at inflection: Multiple comparisons<br>Induced vs uninduced samples			
**Treatment 1**	**Treatment 2**	**P-Value**	**Significance (α 0.05)**
**Control (pH = 6.71)**			
Induced	Uninduced	0.378	ns
**Control (pH = 7.04)**			
Induced	Uninduced	0.056	ns
**BA media (pH = 6.71)**			
Induced	Uninduced	0.598	ns
**BA media (pH = 7.04)**			
Induced	Uninduced	0.49	ns

Multiple comparisons between induced samples grown from cultures previously exposed to BAs and uninduced from cultures grown in non-supplemented VDMP. Cultures grown in (non-supplemented VDMP media, original pH 6.71) and biogenic amine (BA) media (original pH). An additional media of each was prepared and its pH adjusted to mimic its supplemented/non-supplemented counterpart.

Table S5. Effect of amine supplemented media on doubling time.

Doubling time: Multiple comparisons<br>Induced vs uninduced samples			
**Treatment 1**	**Treatment 2**	**P-Value**	**Significance (α 0.05)**
**Induced**			
Control (pH = 6.71)	Control (pH = 7.04)	≤ 0.0001	****
Control (pH = 6.71)	BA media (pH = 6.71)	≤ 0.0001	****
Control (pH = 6.71)	BA media (pH = 7.04)	≤ 0.0001	****
Control (pH = 7.04)	BA media (pH = 6.71)	≤ 0.0001	****
Control (pH = 7.04)	BA media (pH = 7.04)	≤ 0.0001	****
BA media (pH = 6.71)	BA media (pH = 7.04)	≤ 0.0001	****
**Uninduced**			
Control (pH = 6.71)	Control (pH = 7.04)	≤ 0.0001	****
Control (pH = 6.71)	BA media (pH = 6.71)	≤ 0.0001	****
Control (pH = 6.71)	BA media (pH = 7.04)	≤ 0.0001	****
Control (pH = 7.04)	BA media (pH = 6.71)	≤ 0.0001	****
Control (pH = 7.04)	BA media (pH = 7.04)	≤ 0.0001	****
BA media (pH = 6.71)	BA media (pH = 7.04)	≤ 0.0001	****

Multiple comparisons between control group (non-supplemented VDMP media, original pH 6.71) and Biogenic amine (BA) media (original pH). An additional media of each was prepared and its pH adjusted to mimic its supplemented/non-supplemented counterpart. Induced samples were grown from cultures previously exposed to BAs and uninduced from cultures grown in non-supplemented VDMP.

Table S6. Effect of previous amine exposure on doubling time.

Doubling time: Multiple comparisons<br>Induced vs uninduced samples			
**Treatment 1**	**Treatment 2**	**P-Value**	**Significance (α 0.05)**
**Control (pH = 6.71)**			
Induced	Uninduced	0.724	ns
**Control (pH = 7.04)**			
Induced	Uninduced	0.033	*
**BA media (pH = 6.71)**			
Induced	Uninduced	0.593	ns
**BA media (pH = 7.04)**			
Induced	Uninduced	0.062	ns

Multiple comparisons between induced samples grown from cultures previously exposed to BAs and uninduced from cultures grown in non-supplemented VDMP. Cultures grown in (non-supplemented VDMP media, original pH 6.71) and biogenic amine (BA) media (original pH). An additional media of each was prepared and its pH adjusted to mimic its supplemented/non-supplemented counterpart.

Table S7. Effect of amine supplemented media on logistic area under the curve.

Area under the curve (AUC): Multiple comparisons<br>Induced vs uninduced samples			
**Treatment 1**	**Treatment 2**	**P-Value**	**Significance (α 0.05)**
**Induced**			
Control (pH = 6.71)	Control (pH = 7.04)	≤ 0.0001	****
Control (pH = 6.71)	BA media (pH = 6.71)	≤ 0.0001	****
Control (pH = 6.71)	BA media (pH = 7.04)	≤ 0.0001	****
Control (pH = 7.04)	BA media (pH = 6.71)	≤ 0.0001	****
Control (pH = 7.04)	BA media (pH = 7.04)	≤ 0.0001	****
BA media (pH = 6.71)	BA media (pH = 7.04)	≤ 0.0001	****
**Uninduced**			
Control (pH = 6.71)	Control (pH = 7.04)	≤ 0.0001	****
Control (pH = 6.71)	BA media (pH = 6.71)	≤ 0.0001	****
Control (pH = 6.71)	BA media (pH = 7.04)	≤ 0.0001	****
Control (pH = 7.04)	BA media (pH = 6.71)	≤ 0.0001	****
Control (pH = 7.04)	BA media (pH = 7.04)	≤ 0.0001	****
BA media (pH = 6.71)	BA media (pH = 7.04)	≤ 0.0001	****

Multiple comparisons between control group (non-supplemented VDMP media, original pH 6.71) and Biogenic amine (BA) media (original pH). An additional media of each was prepared and its pH adjusted to mimic its supplemented/non-supplemented counterpart. Induced samples were grown from cultures previously exposed to BAs and uninduced from cultures grown in non-supplemented VDMP.

Table S8. Effect of previous amine exposure on logistic area under the curve.

Area under the curve (AUC): Multiple comparisons<br>Induced vs uninduced samples			
**Treatment 1**	**Treatment 2**	**P-Value**	**Significance (α 0.05)**
**Control (pH = 6.71)**			
Induced	Uninduced	0.045	*
**Control (pH = 7.04)**			
Induced	Uninduced	0.029	*
**BA media (pH = 6.71)**			
Induced	Uninduced	0.68	ns
**BA media (pH = 7.04)**			
Induced	Uninduced	0.032	*

Multiple comparisons between induced samples grown from cultures previously exposed to BAs and uninduced from cultures grown in non-supplemented VDMP. Cultures grown in (non-supplemented VDMP media, original pH 6.71) and biogenic amine (BA) media (original pH). An additional media of each was prepared and its pH adjusted to mimic its supplemented/non-supplemented counterpart.
